# Total Phenolic Contents and Antioxidant Activities of Different Extracts and Fractions from the Aerial Parts of* Artemisia biennis *Willd

**Published:** 2014

**Authors:** Tayyebe Hatami, Sayyed Ahmad Emami, Sayyed Shahram Miraghaee, Mahdi Mojarrab

**Affiliations:** a *Novel Drug Delivery Research Center, School of Pharmacy, Kermanshah University of Medical Sciences, Kermanshah, Iran.*; b *Student Research Committee, Kermanshah University of Medical Science, Kermanshah, Iran.*; c Department of Pharmacognosy, School of Pharmacy, Mashhad University of Medical Sciences, Mashhad, *Iran. *

**Keywords:** *Artemisia biennis*, Total phenolic content, Antioxidant activity

## Abstract

Total phenolic contents (TPC) of five different extracts (petroleum ether, dichloromethane, ethyl acetate, ethanol and ethanol-water) of *Artemisia biennis *Willd were measured in this work. The antioxidant activity was investigated by three different methods: β-carotene bleaching (BCB) test, 2,2-diphenyl-1-picrylhydrazyl (DPPH) radical scavenging method and ferrous ion chelating (FIC) assay. Among all the extracts analyzed, the hydroethanolic extract exhibited a significantly higher phenolic content and antioxidant activity than other samples. Vacuum liquid chromatography of this extract yielded seven fractions (A to G) which were subjected to all aforementioned experiments. The highest total phenolic content and free radical scavenging activites were present in the same sample (Fraction D) but the only statistically significant correlation between TPC and EC_50_ values was observed for BCB.

## Introduction

Free radicals are extremely unstable and reactive compounds generated in the body during normal metabolic function or due to exposure to exogenous factors (1-2). Superoxide, hydroxyl and peroxide radicals, hydrogen peroxide and singlet oxygen which are categorized as reactive oxygen species (ROS) are known to cause oxidative damage (2-3). Oxidative damage plays a significantly pathological role in the initiation and/or progression of human diseases, such as atherosclerosis, myocardial and cerebral ischemia, inflammatory injury, diabetes, cancer, rheumatoid arthritis, cardiovascular diseases as well as in the aging process (4-5). Enough amounts of exogenous antioxidants are able to reduce the harm of ROS to the human body. These compounds can delay or inhibit the oxidative damage of proteins, nucleic acids and lipids caused by free radical- induced oxidative stress (6-8). The oxidation process can be interfered by chelating the catalytic metals and also by acting as free radical scavengers (9).

Nowadays, much attention is paid to use natural compounds because some synthetic antioxidants such as butylated hydroxyanisole (BHA), butylated hydroxytoluene (BHT) and *tert*-butylhydroquinone (TBHQ) are supposed to be responsible for carcinogenesis and liver damage in laboratory animals (10-12). Plants are potential sources of natural antioxidants such as ascorbic acid, tocopherol, carotenoids, flavonoids and phenolic acids (13-14). In many studies it has been revealed that there is a direct relationship between antioxidant activity and phenolic content of plant extracts (15-17). Many reported biological effects such as antioxidant, antimutagenic, anticancer and anti-inflammatory activities have been attributed to the presence of phenolic compounds which are widely distributed in plants (18-19). Nitrogen containing compounds are the other class of secondary metabolites which effectively prevent lipid peroxidation and inhibit protease and RNase activity seen as an outcome of oxidative stress in plants (20). They have shown significant effects on maintainability of intracellular Ca^2+^ homeostasis due to similar actions of polyamines in human body (21). Polysaccharide fractions (22-23) and terpenoids (24-25) are regarded as natural antioxidants with different mechanisms including scavenging activity of free radicals, reducing power and metal chelating ability 

*Artemisia biennis* Willd. (Compositae) which is called “Dermaneye dosaaleh” and “Dermaneye mortafa” in persian language, is one of 34 *Artemisia* species growing wildly in Iran (26). The first study on the composition of the essential oil of* A.biennis *grown in Iran has revealed the presence of alpha-pinene (10.2%), 1,8-cineole (10.1%), artemisia ketone (11.4%) and camphor (24.6%) as the main components (27). Volatiles from the aerial parts of *A. biennis* from western Canada were also identified as in (Z)-beta-ocimene (34.7%), (E)-beta-farnesene (40.0%), the acetylenes (11.0%) and (Z)- and (E)-en-yn-dicycloethers. Additionally, weak antioxidant and free radical scavenging activities and strong effect against *Cryptococcus neoformans, Fonsecaea pedrosoi* and *Aspergillus niger* were found for the oil (28). Dose-dependent and strong inhibition of cancer cell growth by different fractions obtained from seven *Artemisia *species has been reported. In this study, dichchloromethane fraction of *A.biennis* showed the highest cytotoxicity on the cervical cancer cell line (29). The ethanolic extract of *Artemisia biennis *along with similar extracts of ten other *Artemisia *species showed significant effects on *in-vitro* leishmanicidal activity (30). The aim of the present work is to undertake an investigation of the antioxidant activity and phenolic content of different extracts and fractions of *Artemisia biennis *Willd. grown in Iran.

## Exprimental

*Chemicals*

β- Carotene and 1,1-diphenyl-2-picryl-hydrazyl (DPPH) were purchased from Sigma- Aldrich. Linoleic acid, gallic acid, ferrous chloride, sodium carbonate, dimethyl sulfoxide (DMSO), chloroform, Tween^®^ 40, Folin-Ciocalteu’s phenol reagent, ethylenediaminetetraacetic acid (EDTA), butylated hydroxytoluene (BHT), LiChroprep^®^ RP-18 (15-25 µm) were purchased from Merck, ascorbic acid from VWR, ferrozine iron reagent from Acros Organics and all the solvents used for extraction from Scharlau. 

*Plant material*

Aerial parts of *Artemisia biennis *Willd. were collected from Zoshk (Razavi Khorasan province, Iran) in September 2010. The plant was compared with voucher specimen (voucher specimen No. 12570) deposited at the Department of Pharmacognosy, Faculty of Pharmacy, Mashhad University of Medical Sciences, Mashhad, Iran.

*Preparation of extracts and fractions*

The dried powdered aerial parts (800 g) of* Artemisia biennis *were extracted with petroleum ether (40-60), dichloromethane, ethyl acetate, ethanol and ethanol-water (1:1 v/v) respectively (Sequential maceration with ca. 3×8 L of each solvent). The extracts were filtrated with filter paper and dried using rotary evaporator at a reduced pressure at a temperature below 45 ^0^C to yield 42.2, 57.8, 3.7, 11.4 and 79.5 g of each extract, respectively. 45 g of the most active extract (hydroethanolic) was subjected to a vacuum liquid chromatography (VLC) system (reversed-phase RP-18 [25-40 µm], 225 g) with H_2_O containing increasing amounts of MeOH (5%, 10%, 20%, 40%, 60%, 80% and 100%) to give seven fractions (A, B, C, D, E, F and G) respectively (Table 1).

**Table 1:** Antioxidant performance and total phenolic contents of the extracts/fractions from *A.biennis.*

**Sample**	**Extraction/ fractionation yield (g)**	**EC**_50_ **(µg/ mL)**			**TPC** **(mg GAE /g)**
		**DPPH assay**	**FIC assay**	**BCB assay**	
PE	42.20	1314.86 ±37.01	258.17 ± 27.71	283.37 ± 4.31	0.19 ± 0.33
DCM	57.80	452 ± 0.02	343.27 ± 10.13	313.18 ±6.25	8.65 ± 2.10
EA	3.70	74.20 ± 1.17	578.71 ± 37.17	212.20 ± 6.71	66.95 ± 0.56
EtOH	11.40	67.39 ± 2.72	269.85 ± 10.69	189.63 ± 7.10	107.02 ± 4.23
EtOH/Wt	79.50	44.05 ± 0.42	132.47 ± 5.33	132.63 ± 4.43	122.21 ± 1.85
Fr. A	19.35	214.41 ± 2.74	22.79 ± 1.34	58.44 ± 0.72	12.33 ± 0.48
Fr. B	6.89	51.50 ± 0.19	30.95 ± 1.55	71.86 ± 2.97	69.15 ± 2.25
Fr.C	2.15	22.01 ± 0.18	54.55 ± 4.97	53.27 ± 16.79	289.13 ± 4.20
Fr. D	4.16	14.98 ± 0.14	66.07 ± 3.09	17.55 ± 3.01	338.61 ± 6.67
Fr. E	3.46	23.64 ± 0.69	115.66 ± 2.09	18.56 ±5.30	318.16 ± 3.59
Fr. F	1.88	27.69 ± 0.45	242.42 ± 7.83	158.07± 1.96	62.67 ± 1.92
Fr. G	1.35	194.74 ± 9.16	180.47± 2.69	245.02 ± 13.46	17.48 ± 10.83
BHT	---	4.88±0.57	---	0.458±0.07	---
Vit C	---	3.66±0.28	---	---	---
EDTA	---	---	18.00 ± 3.02	---	---
Quercetin	---	---	87.24 ± 3.94	---	---

*Total phenolic contents*

The total phenolic content (TPC) was determined by the Folin–Ciocalteu method (31-32) with some modification. 500 µL of different concentrations -depending on solubility- of extracts or fractions in water was mixed with 2.5 mL of Folin- Ciocalteu reagent (0.2 N). After 5 min 2 mL of Na_2_CO_3 _solution (75 g/L) was added, after 120 min standing in dark, the optical density was measured at 760 nm against a blank. The total phenolic contents were calculated on the basis of the calibration curve of gallic acid and expressed as gallic acid equivalents (GAE), in milligrams per gram of the sample.

*DPPH radical scavenging activity*

The radical scavenging activity was assayed using the method of Hatano *et al.* (33) with slight modifications. Briefly, 0.2 mM solution of DPPH in methanol was prepared and 1.5 mL of this solution was added to the equal volume of each of test samples dissolved in methanol at different concentrations. The mixture was shaken vigorously and maintained in dark for 30 min. Then, the absorbance was measured at 517 nm against a blank. Ascorbic acid and butylated hydroxyanisole (BHA) were used as standard references. The scavenging activity was calculated using the formula:

scavenging activity (%)= [(A_517_ of control- A_517 _of sample)/ A_517_ of control]×100.

*Metal chelating activity*

The chelating activity of extracts and fractions for ferrous ions Fe^2+ ^was determined by the ferrous iron– ferrozine complex method (34) with some modification. Briefly, 25µL of FeCl_2 _solution (2 Mm) was added to a mixture containing 1.5 mL of H_2_O and 2 mL of the test samples in methanol at different concentrations. The reaction was initiated by adding 50 µL of ferrozine solution (5 mM) after 30 seconds. The mixture was shaken well and incubated for 10 min at room temperature. Absorbance of the solution was then measured at 562 nm. Quercetin and EDTA were used as positive controls. The ability of the extracts and fractions to chelate ferrous ion was calculated using the equation described above for DPPH.

*Inhibition of β-carotene bleaching*

Antioxidant activity of the extracts and fractions was determined according to a slightly modified version of the β-carotene bleaching method (35). In this study 5 mg of β-carotene was dissolved in 10 mL of chloroform. 750 µL of β-carotene solution, 33 µL of linoliec acid and 225 mg of Tween 40 were mixed. The solvent was completely removed using a rotary evaporator. Then 75 ml of oxygenated distilled water was added and the mixture was emulsified for 15 min in a sonicator to form emulsion A. Aliquots of 3.5 mL of this emulsion were transferred into a series of stopper test tubes containing 1 ml of samples dissolved in DMSO or water in various concentrations. Optical density (OD) at 470 nm was determined for all samples immediately (t=0) and at the end of the time (t=120). A second emulsion was also prepared and used as blank to zero the spectrophotometer. This emulsion consisted of 50 mL of oxygenated water, 22 µL of linoleic acid and 150 mg of tween 40. The percentage inhibition was calculated according to the following formula: 

% inhibition = [(A_A(120) _– A_C(120)_) / (A_C(0) _– A_C(120)_)] ×100 

Where A_A(120) _is the absorbance of the sample at t=120 min, A_C(120)_ is the absorbance of the control at t=120 min, and A_C(0) _ is the absorbance of the control at t=0 min.

*Statistical analysis*

The experimental results were performed in triplicate. The data were recorded as mean ± standard deviation and analyzed by SPSS (version 16 for Windows Xp). Non- parametric Friedman test was performed by following the procedures and p < 0.05 was regarded as significant. Pearson’s correlation coefficients (r) between total phenolic contents of the samples and calculated EC50 values in each antioxidant assay were determined.

## Results and Discussion

*Total phenolic contents (TPC) of different extracts and fractions*

Phenolics which exist naturally in an approximated number of 8000, share the identical prevalent structure composed of an aromatic hydroxyl nucleus (36). So far, plant phenolics form one of the main groups of compounds working as primary antioxidants or free radical scavengers. Plant polyphenols are effective as singlet oxygen scavengers, reducing agents and hydrogen atom donators. (36- 37). For this reason, it is logical to ascertain their total amount in the prepared extracts and fractions of *Artemisia biennis* . Feasible intervention from other readily oxidized compounds in the plant materials and heterogeneousness of natural phenolics has led to introduction of several methods for determination of total phenolics. In most cases, Folin-Ciocalteu method has been found preferable as compared to the others (38). In this study, a blue-coloured solution -due to the presence of phospho molybdic-phosphotungstic-phenol complex- was produced when the active extracts or fractions reacted with Folin-Ciocalteau reagent in an alkaline medium. The content of phenolics was calculated from the regression equation of the calibration curve (R^2^ =0.989, *y *= 0.009*x* + 0.0464), expressed in GAE as milligrams per gram of the extract or fraction (mg GAE/g extract or fraction). The total phenolic content of the samples showed large variations, between 0.19 ± 0.33 and 338.61 ± 6.67 mg GAE/g extract (Table 1). Based on the results, the extracts contained a mixture of phenolic compounds at different levels in the following order: hydroethanol > ethanol > ethyl acetate> dichloromethane> petroleum ether. Three fractions (C, D and E) of the hydroethanolic extract had a remarkably high total phenolic content. Fraction D contained the highest total phenol content (338.61 ± 6.67 mg GAE/g fraction), followed by fractions E (318.16 ± 3.59 mg GAE/g fraction) and C (289.13 ± 4.20 mg GAE/g fraction).

*Antioxidant activities of A. biennis extracts and derived fractions from (hydroethanolic* *(* *extract*

*DPPH assay*

Comparatively stable organic radical DPPH has been broadly utilized in determination of the antioxidant activity of different plant extracts as well as purified compounds (39, 40). The ability of antioxidants for DPPH radical scavenging is supposed to be due to their hydrogen donating property (41). After Acceptance of an electron or a hydrogen atom, a stable diamagnetic molecule will emerge which will result in vanishing the absorption band at 517 nm. The radical scavenging activity of the samples corresponds to the remaining DPPH in an inverse manner (42). 

With the exception of petroleum ether and dichloromethane extracts and Fractions A and G, all the extracts and fractions showed moderate to good inhibitory performance with respect to the DPPH radical. The highest activity was obtained from the fraction D, with the EC_50_ value of 14.98 ± 0.14 µg/mL, followed by the fractions C and E with the EC_50_ values of 22 .01

**Figure 1:**
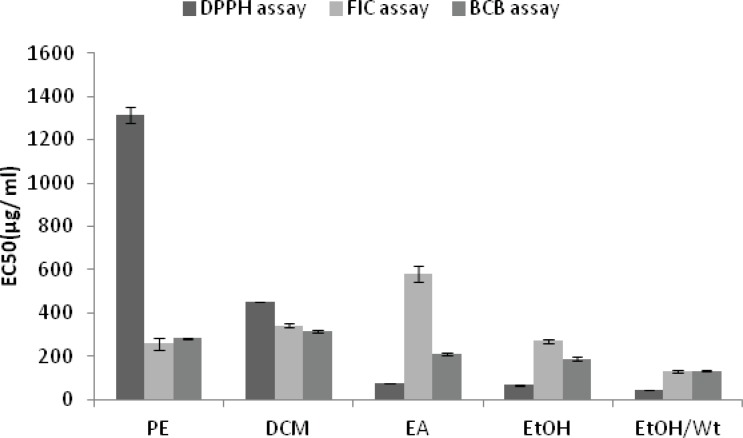
Antioxidant activity of *A.biennis* extracts from petroleum ether (PE), dichloromethane (DCM), ethyl acetate (EA), ethanol (EtOH) and ethanol/water (EtOH/Wt).

**Figure 2:**
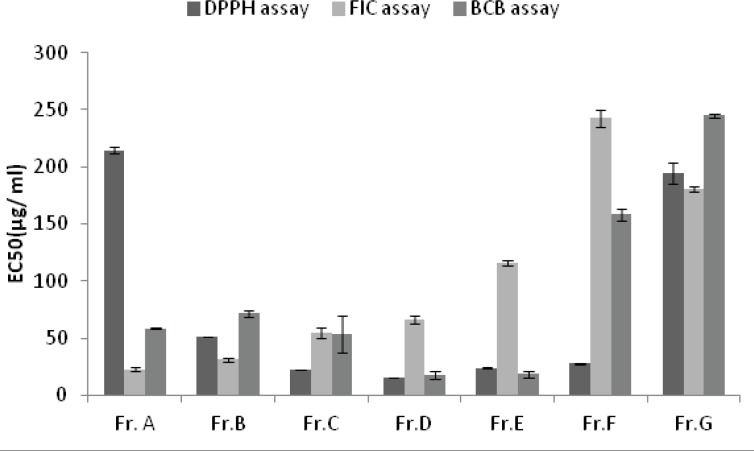
Antioxidant activity of different fractions from hydroethanolic extract of *A.biennis*

 ± 0.18 and 23.64 ± 0.69 µg/mL , respectively (Figure 1 and 2).

*Ferrous ion chelating (FIC) assay*

Fe^2+^ ion is regarded as the most powerful pro-oxidant among various species of metal ions (43). Ferrous ion chelating activity of an antioxidant could prohibit free radical generation and resultant oxidative damage. Fe^2^^+^ can quantitatively form complexes with Ferrozine. Presence of chelating agents results in the disruption of complex formation which is followed by decolorization of the solution. So, measurement of reduction in color intensity permits the estimation of the chelating activity of the sample (44).

 All the extracts except the last one did not show any remarkable colour changes, although decreases in absorbance readings were recorded. Compared to the results of positive controls, five fractions (A to E) had good ability to chelate metal ion. The highest ferrous ion chelating effect among the samples was shown by fraction A, with the EC_50_ value of 22.79 ± 1.34 µg/mL followed by the fractions B and C with the EC_50_ values of 30.95 ± 1.55 µg/mL and 54.55 ± 4.97 µg/mL , respectively (Figures 1 and 2).

*β-* *carotene bleaching (BCB) assay*

In the BCB assay, the oxidation of linoleic acid produces free radicals due to the removing of hydrogen atom from diallylic methylene groups of linoleic acid (45). The highly unsaturated β- carotene then will be oxidized by the generated free radical. Degradation of the orange coloured chromophore of β- carotene could be monitored spectrophotometrically. However, the presence of antioxidant constituents could prevent the bleaching of β-carotene because of their ability to neutralize the free radicals (46, 47). 

Figures 1 and 2 show the inhibitory activity of *A.biennis* extracts and derived fractions on β-*carotene bleaching. *Fraction D showed the best inhibitory performance, with an EC_50_ value of 17.55 ± 3.01 μg/mL while dichloromethane extract (EC_50_= 313.18 ± 6.25 μg/mL) exhibited the lowest.

*Statistical analysis*

Pearson's correlation coefficients between TPC and obtained EC_50_s for DPPH, FIC and BCB assays took the values of -0.474, -0.395, and -0.741 respectively. The results showed lowest correlation between TPC of the samples and their ability to chelate ferrous ions. There was no significant correlation between DPPH radical scavenging activities of the samples and TPC as well. A significant correlation between TPC and the ability of the samples to inhibit the bleaching of β-*carotene was observed. The results of Friedman test showed none of the assays is superior in screening the samples for their antioxidant ability.*

## Conclusion

Total phenolic contents of the most active fractions in FIC method were relatively low. For this reason, it could be concluded that there are some other types of phytochemicals like terpenoides and/or polysaccharides responsible for acting as secondary antioxidants. Isolation and structure elucidation of the components seems to be necessary as the following step. Free radical scavenging activities of the samples showed better correlation to their total phenolic contents as it was clarified by the Pearson's correlation coefficients. In general, the stronger antioxidant activities of *Artemisia biennis *hydroethanolic extract and some of its derived fractions in comparison with the other samples could be attributed to their higher content of phenolic compounds.
